# TDF and quantitative ultrasound bone quality in African patients on second line ART, ANRS 12169 2LADY sub-study

**DOI:** 10.1371/journal.pone.0186686

**Published:** 2017-11-08

**Authors:** Firmin Nongodo Kabore, Sabrina Eymard-Duvernay, Jacques Zoungrana, Stéphanie Badiou, Guillaume Bado, Arsène Héma, Assane Diouf, Eric Delaporte, Sinata Koulla-Shiro, Laura Ciaffi, Amandine Cournil

**Affiliations:** 1 Department of Clinical Research, Centre MURAZ, Bobo-Dioulasso, Burkina Faso; 2 Unité Mixte Internationale 233, Institut de Recherche pour le Développement, U1175-INSERM, University of Montpellier, Montpellier, France; 3 Department of Infectious Diseases, University Hospital Souro Sanou, Bobo-Dioulasso, Burkina Faso; 4 Biochemistry Department, University Hospital, Montpellier, France; 5 Department of Hospital Hygiene, University Hospital Souro Sanou, Bobo-Dioulasso, Burkina Faso; 6 Centre Régional de Recherche et de Formation (CRCF), Dakar, Senegal; 7 Department of Infectious Diseases, University Hospital, Montpellier, France; 8 Servives des maladies infectieuses, Yaoundé central hospital, Yaoundé, Cameroon; 9 Faculté de Médecine et des Sciences Biomédicales, University of Yaoundé 1, Yaoundé, Cameroon; Rush University, UNITED STATES

## Abstract

**Background:**

Bone demineralization, which leads to osteoporosis and increased fracture risk, is a common metabolic disorder in HIV-infected individuals. In this study, we aimed to assess the change in bone quality using quantitative ultrasound (QUS) over 96 weeks of follow-up after initiation of second-line treatment, and to identify factors associated with change in bone quality.

**Methods and findings:**

In a randomized trial (ANRS 12169), TDF and PI-naïve participants failing standard first-line treatment, from Burkina Faso, Cameroon, and Senegal were randomized to receive either TDF/FTC/LPVr, ABC/ddI/LPVr or TDF/FTC/DRVr. Their bone quality was assessed using calcaneal QUS at baseline and every 24 weeks until week 96. Stiffness index (SI) was used to measure bone quality.

Out of 228 participants, 168 (74%) were women. At baseline, median age was 37 years (IQR: 33–46 years) and median T-CD4 count was 199 cells/μl (IQR: 113–319 cells/μl). The median duration of first-line antiretroviral treatment (ART) was 52 months (IQR: 36–72 months) and the median baseline SI was 101 (IQR: 87–116). In multivariable analysis, factors associated with baseline SI were sex (β = -10.8 [-18.1,-3.5] for women), age (β = -8.7 [-12.4,-5.1] per 10 years), body mass index (BMI) (β = +0.8 [0.1,1.5] per unit of BMI), and study site (β = +12.8 [6.5,19.1] for Cameroon). After 96 weeks of second-line therapy, a reduction of 7.1% in mean SI was observed, as compared with baseline. Factors associated with SI during the follow-up were similar to those found at baseline. Exposure to TDF was not associated with a greater loss of bone quality over time.

**Conclusion:**

Bone quality decreased after second-line ART initiation in African patients independently of TDF exposure. Factors associated with bone quality include age, sex, baseline BMI, study site, and duration of follow-up.

## Introduction

Bone demineralization, which leads to osteoporosis and increased fracture risk, is a common metabolic disorder in HIV-infected individuals, with up to three times the prevalence of osteoporosis and 30–70% higher occurrence of fracture as compared with HIV-uninfected controls [1,2]. The causes of low bone mineral density (BMD) in patients with HIV infection are likely multifactorial, involving traditional risk factors such as aging, low body mass index (BMI), smoking, female gender; HIV infection; and exposure to antiretroviral treatments (ART) [3–9]. Several studies have reported that a lower BMD is present in patients on ART as compared with BMD levels in untreated patients [8,10]. Certain randomized studies have shown that tenofovir disoproxil fumarate (TDF), an antiretroviral widely used in low- and middle-income countries, is associated with greater bone loss, but other observational studies have failed to confirm this association [11–16]. The aging of people living with HIV (PLHIV) raises the problem of bone health in this population. The number of PLHIV aged 50 years and over is increasing, and presently, there are more than 4 million patients, with half of them living in sub-Saharan Africa [17]. However, limited data exists regarding BMD levels in HIV-infected patients living in sub-Saharan Africa. The ANRS 12169 2LADY randomized trial (NCT00928187), conducted in three African cities, which aimed to compare efficacy and safety of three second-line regimens on TDF-naïve patients, provided an opportunity to assess the impact of TDF-based regimen on bone loss [18]. In the trial sub-study ANRS 12250 METABODY, bone quality was assessed via the use of calcaneal quantitative ultrasound (QUS) as an alternative to dual-energy X-ray absorptiometry (DXA), which is not available in most African settings [7,19,20]. This current study assessed the change in bone quality during 96 weeks of follow-up after second-line treatment was initiated and also identified the associated factors.

## Materials and methods

### Study design and participants

ANRS 12250 METABODY is an observational longitudinal sub-study nested in the ANRS 12169 2LADY trial. The objective of METABODY was to describe the morphological changes and metabolic disorders (i.e., metabolic syndrome and cardiovascular and bone changes) in HIV-positive patients who failed first-line antiretroviral treatment, and their evolution on second-line ART.

ANRS 12169 2LADY is a multicenter randomized, open-label, phase III trial that compared efficacy and safety of three second-line ART combinations in three African cities: Bobo Dioulasso (in Burkina Faso), Dakar (in Senegal), and Yaoundé (in Cameroon). HIV-1 positive patients aged ≥ 18 years and TDF-naïve who had virologically failed a non-nucleoside reverse transcriptase inhibitor (NNRTI)-based first-line ART after at least six months of administration were enrolled from January 2010 to September 2012 and randomized to receive tenofovir/emtricitabine + lopinavir/ritonavir or abacavir + didanosine + lopinavir/ritonavir or tenofovir/emtricitabine + darunavir/ritonavir (TDF/FTC + LPVr, ABC + ddI + LPVr or TDF/FTC + DRVr) [18]. All patients enrolled in the 2LADY trial from 2011 to 2012 were offered an opportunity to participate in METABODY.

### Clinical and biological data collection

Clinical and biological data (e.g., general condition, weight, blood pressure, creatinine, glycemia, cholesterolemia, insulinemia, blood count, 25-hydroxy vitamin D (25-OH vitamin D), parathyroid hormone (PTH), CD4 count, viral load, triglyceridemia, SI, physical activity, hormonal contraception use, glucocorticoid use, dysmenorrhea and diagnosis of menopause) were collected at baseline and every 24 weeks. Participant physical activity was assessed with a short frequency questionnaire, in which “regular physical activity” was defined as moderate activity occurring each day or strenuous activity occurring at least twice a week. Some laboratory tests related to bone metabolism that were not available at the study sites were centralized in the laboratory of Montpellier University Hospital (in France). Measurements of insulin, PTH and 25-OH vitamin D levels were performed on frozen serum (-80°C) using chemiluminescent method on a Cobas 6000/e601 analyzer (Roche Diagnostics, Indianapolis, IN, USA). The three tests were included in the routine methods of lab. Between-run imprecision was <5% for insulin and PTH, and was <11.5% for 25-OH vitamin D.

### Bone quality outcomes measures

Bone quality was assessed by calcaneal QUS (Achilles, GE Healthcare, Little Chalfont, UK) at baseline and every 24 weeks for 96 weeks. QUS parameters included broadband ultrasound attenuation (BUA), a measure of the frequency-dependent attenuation of ultrasound, and speed of sound (SOS), which reflected the transmission velocity of ultrasound passing through soft tissue and bone tissue. Stiffness index (SI) calculated by the device (SI = 0.67xBUA + 0.28xSOS– 420) was used to measure bone quality because it had been shown to be better predictor of fracture than BUA and SOS [19,21]. At each visit, three repeated measurements of bone quality were realized for each participant and the average of the two closest SI measures was used for the analysis.

### Ethics

Written informed consent was obtained from all participants in this study. Study protocol was approved by the “National Ethics Committee” for Cameroon (07/26/2010), the “Comité National d’Ethique pour la Recherche en Santé” for Sénégal (10/07/2010), the “Comité d’Ethique pour la Recherche en Santé” for Burkina Faso (11/19/2010), and the study was conducted in accordance with the Declaration of Helsinki and Good Clinical Practices.

### Statistical analysis

Analyses included available data from all participants enrolled in METABODY sub-study and those who completed a baseline measure of bone quality. For women who became pregnant during the study, bone quality measures from the date of pregnancy were not included in the longitudinal analyses.

The main dependent variable was mean SI. The effects of TDF were assessed by comparing TDF-containing treatment arms (TDF/FTC + LPVr or DRVr) to a TDF-sparing treatment arm (ABC + ddI + LPVr). Demographic factors (i.e., study site, sex, age, year of inclusion); HIV related factors (i.e., WHO clinical stage, CD4 count, viral load, first-line ART duration, Efavirenz (EFV)-based first line treatment); and other factors involved or possibly involved in bone metabolism (i.e., regular physical activity, metabolic syndrome, body mass index, menopause, contraception with Depo-Provera® (Pfizer, New York, NY, USA), hepatitis B antigen-positive, 25-OH vitamin D, PTH) were also assessed for association with bone quality outcomes.

In a baseline characteristics comparison, Student’s t-test was used for continuous variables, and Pearson chi-square or Fisher’s exact test was used for categorical ones. Specific factors associated with baseline SI were identified using linear regression models, and mixed linear models with random effects were used to determine associated factors during participant follow-up [22]. Furthermore, to account for women-specific characteristics (i.e., menopause, amenorrhea and/or use of hormonal contraception), additional analyses were performed in the women sub-group only. Ultimately, our study demonstrated enough power (80%) to show a difference of 4.1 SI units (representing about 0.2 SD) between treatments given with and without TDF (which was calculated using the R package " Longpower " for linear mixed models based on the formula due to Diggle) [23]. STATA version 14 (StataCorp., College Station, Texas, USA) was used for the statistical analyses.

## Results

### Baseline characteristics

From March 2011 to October 2012, 273 participants were enrolled in the METABODY study; 228 subjects with baseline SI measurements were included in the present analysis. Demographic and osteoporosis-related characteristics of participants at baseline are shown in **Table 1**. The population was predominantly female (73.7%), and most of the participants were recruited in Cameroon (69.3%). Their median age was 37.3 years (Interquartile range (IQR): 32.9–46.2 years), and 14.9% of participants were at least 50 years old. Participants were comparable in the three treatment arms with respect to baseline characteristics. Patients who started TDF-containing treatment were also comparable to those who received TDF-sparing treatment, with respect to baseline characteristics **(Table 1)**.

**Table 1:** Baseline characteristics of participants and osteoporosis’ classic risk factors.

	TDF/FTC+LPVr or DRVr (n = 156)	ABC+ddI +LPVr (n = 72)	p-value
Age (years)	37.3 (32.8–44.7)	37.3 (33.4–48.0)	0.14
Age ≥ 50 years	21 (13.5%)	13 (18.1%)	0.37
Female	118 (75.6%)	50 (69.4%)	0.32
Study site			0.58
Burkina Faso	34 (21.8%)	17 (23.6%)	
Senegal	15 (9.6%)	4 (5.6%)	
Cameroun	107 (68.8%)	51 (70.8%)	
Year of inclusion in METABODY			0.29
2011	97 (62.2%)	50 (69.4%)	
2012	59 (37.8%)	22 (30.6%)	
BMI (kg/m^2^)	24.1 (21.9–26.3)	23.5 (21.3–28.3)	0.39
Smoking	7 (4.5%)	1 (1.4%)	0.44
Regular alcohol consumption	6 (3.9%)	1 (1.4%)	0.44
Regular physical activity ^**a**^	101 (64.7%)	50 (69.4%)	0.49
Diabetes	0	2 (2.8)	0.10
High blood pressure	11 (7.1%)	9 (12.5%)	0.18
Glucocorticoid use	2 (1.3%)	3 (4.2%)	0.18
Secondary osteoporosis	0	1 (1.4%)	0.32
Fracture history	8 (5.1%)	4 (5.6%)	1.00
Hip fracture of parent	3 (1.9%)	3 (4.2%)	0.38
Metabolic syndrome ^**b**^	15 (9.6%)	11 (15.3%)	0.21
HOMA ^**c**^ *	1.4 (0.9–2.3)	1.5 (1.1–2.8)	0.72
FRAX score ^**d**^	0 (0–0.1)	0 (0–0.1)	0.35
FRAX > 0%	66 (42.3%)	35 (48.6%)	0.37
Vitamin D (ng/ml) *	34.4 (27.0–42.4)	35.5 (26.6–43.9)	0.70
Vitamin D rate < 30 ng/ml *	54 (36.0%)	22 (31.4%)	0.44
PTH (pg/ml) *	40.4 (31.8–52.9)	42.3 (30.3–54.5)	0.81
Menopause	15 (12.7%)	10 (20.0%)	0.23
Current Dépo-provera using	5 (3.2%)	2 (2.8%)	1.00
Amenorrhea	6 (3.9%)	2 (2.8%)	1.00
eGFR^**e**^ < 60 ml/min/1.73m^2^	5 (3.2%)	3 (4.2%)	0.71
HBs-antigen positive	12 (7.7%)	5 (6.9%)	0.84
Stiffness index	102.3 (86.8–116.5)	99.3 (87.5–113.8)	0.64

Data are median (IQR) or n (%). BMI: body mass index (mass(kg)/height^2^(m)). PTH: parathyroid hormone; HBs-antigen: Hepatitis B surface antigen.

* There were 8 missing data for those variables.

^a^ Regular physical activity: moderate physical activity every day or high physical activity twice a week

^b^Metabolic syndrome according to IDF definition (http://www.idf.org/metabolic-syndrome)

^c^HOMA: Homeostasis Model Assessment of Insulin Resistance (insuline(mUI/l) x glycémie(mmol/l) /22.5)

^d^FRAX: 10-year probability of fracture (http://www.shef.ac.uk/FRAX/)

^e^eGFR: estimated glomerular filtration rate (using Cockroft and Gault formula (male: 1.23 x weight (kg) x (140-age) / creatinine (μmol/l) and female: 1.04 x weight (kg) x (140-age) / creatinine (μmol/l)).

At baseline, the SI intra-individual coefficient of variation between the three measures of bone quality was 5.2% [2.6%, 7.8%]. SI values ranged from 35.0 to 167.5 with a median of 101 (IQR = 87–116). Median SI was 101.3 (IQR: 86.8–115.5) for women, and 99.8 (IQR: 87.0–119.3) for men. Postmenopausal women demonstrated a median SI of 84.5 (IQR: 77.5–100.5) as compared with a median SI of 104.0 (IQR: 91.0–116.5) for premenopausal women. Participants who were older than 50 years of age had a median SI of 87.3 (IQR: 73.5–110.0) as compared with 103.5 (IQR: 90.5–116.5) in those under 50 years of age. No difference between median SI in Burkina Faso (95; IQR: 79.5–104.5) and Senegal (96; IQR: 83–106), (p-value = 0.89) was observed; however, the median SI in Cameroon (106.8; IQR: 90.5–119.5) was higher than those from both Burkina Faso and Senegal (p-value < 0.001).

HIV related characteristics of participants are shown in **Table 2**. The majority (90.8%) were asymptomatic for HIV infection at baseline. Median CD4 count was 199 cells/μl (IQR: 113–319 cells/μl) and median viral load was 4.5 log/ml (IQR: 4.0–5.1). Participants received predominantly (i.e., 75.9%) (zidovudine/lamivudine/nevirapine) AZT/3TC/NVP as a first-line ART. The duration of first-line ART administration ranged from 10.3 months to 114.4 months, with a median of 51.9 months (IQR: 36.1–71.7).

**Table 2:** HIV infection related characteristics at baseline.

	TDF/FTC+LPVr or DRVr (n = 156)	ABC+ddI +LPVr (n = 72)	p-value
WHO stage			0.90
1	140 (89.7%)	67 (93.1%)	
2	10 (6.4%)	4 (5.6%)	
3	5 (3.2%)	1 (1.4%)	
4	1 (0.6%)	0	
Viral load (log/ml)	4.5 (3.9–5.0)	4.6 (4.1–5.1)	0.32
Viral load ≥ 100 000 copies/ml	40 (25.6%)	23 (31.9%)	0.32
CD4 count (cells/μl)	196 (110–333)	204 (130–288)	0.79
CD4 count < 200 cells/μl	80 (51.3%)	35 (48.6%)	0.71
Nadir CD4 count < 200 cells/μl *	123 (83.1%)	60 (85.7%)	0.63
CD4 count at first-line initiation (cells/μl) ^†^	118 (60–188)	144 (73–192)	0.21
CD4 count at first-line initiation < 200 cells/μl ^†^	118 (80.8%)	53 (76.8%)	0.50
First-line ART regimens			0.32
AZT/3TC/NVP	123 (78.9%)	50 (69.4%)	
AZT/3TC/EFV	29 (18.6%)	20 (27.8%)	
d4T/3TC/NVP	3 (1.9%)	2 (2.8%)	
d4T/3TC/EFV	1(0.6%)	0	
First-line ART duration (months)	50.3(34.5–71.1)	58.1(37.5–74.5)	0.24
< 36	42 (29.9%)	15 (20.8%)	0.61
[36–72[	77 (49.4%)	38 (52.8%)	
≥ 72	37 (23.7%)	19 (26.4%)	

Data are median (IQR) or n (%). WHO: world health organization; ART: antiretroviral treatment; AZT: Zidovudine; 3TC: Lamivudine; NVP: Nevirapine; EFV: Efavirenz; d4T: Stavudine

* There were 10 missing data for this variable

^†^ There were 13 missing data for this variable

### Factors associated with Stiffness index at baseline

In multivariable analysis, higher SI was associated with higher BMI and younger age. SI was also significantly higher in males and in patients from Cameroon. Neither CD4 count, viral load, duration of ART, EFV-base first-line ART nor other HIV infection-related factors were associated with SI level **(Table 3)**.

**Table 3:** Factors associated with Stiffness index at baseline, for all participants.

	Univariable			Multivariable		
	β	95% CI	p-value	β	95% CI	p-value
***Demographic factors***						
Female	-0.5	[-6.9; 5.9]	0.88	-10.8	[-18.1; -3.5]	0.004
Age (per 10 years)	-5.3	[-8.1; -2.5]	<0.001	-8.7	[-12.4; -5.1]	<0.001
Cameroun ^a^	12.3	[6.4; 18.2]	<0.001	12.8	[6.5; 19.1]	<0.001
Year of inclusion in METABODY (2011 vs 2012)	5.3	[-0.5; 11.2]	0.07	4.0	[-1.6; 9.7]	0.16
***HIV related factors***						
WHO stage > 1	-3.2	[-12.9; 6.6]	0.53			
WHO stage > 2	-2.6	[-8.3; 3.1]	0.37			
Viral load (log/ml)	0.2	[-3.9; 4.3]	0.91			
Baseline CD4 count < 200 cells/μl	0.3	[-5.4; 5.9]	0.93			
EFV based first-line ART regimen	-4.4	[-11.2; 2.4]	0.20	-3.4	[-9.9; 3.2]	0.31
First-line ART duration (months)			0.08			0.07
< 36	Ref			Ref		
[36–72[	6.9	[0.0; 13.7]		7.5	[0.8; 14.1]	
≥ 72	1.1	[-6.9; 9.1]		2.8	[-5.1; 10.8]	
***Other factors***						
BMI (kg/m^2^)	1.1	[0.5; 1.8]	<0.001	0.8	[0.1; 1.5]	0.03
FRAX ^b^ > 0%	-9.3	[-14.8; -3.7]	0.001	1.7	[-5.2; 8.6]	0.63
Regular physical activity ^c^	-4.7	[-10.7; 1.2]	0.12	0.8	[-5.5; 7.0]	0.81
Vitamin D (ng/ml)	-0.2	[-0.4; 0.1]	0.21	-0.1	[-0.3; 0.1]	0.36
Metabolic syndrome ^d^	3.5	[-5.4; 12.4]	0.44			
HOMA^e^	0.4	[-0.3; 1.0]	0.23	0.3	[-0.3; 0.9]	0.30
eGFR^f^ < 60ml/min/1.173m^2^	-9.8	[-25.1; 5.5]	0.21	4.0	[-11.9; 19.9]	0.62
HBs-antigen positive	-1.1	[-11.9; 9.6]	0.84			

WHO: World Health Organization; EFV: efavirenz; ART: antiretroviral treatment; BMI: body mass index (mass(kg)/height^2^(m)); HBs-antigen: Hepatitis B surface antigen.

^a^ Cameroun (Cameroun vs Burkina Faso and Senegal)

^b^ FRAX: 10-year probability of fracture (http://www.shef.ac.uk/FRAX/)

^c^ Regular physical activity: moderate physical activity every day or high physical activity twice a week

^d^ Metabolic syndrome according to IDF definition (http://www.idf.org/metabolic-syndrome)

^e^ HOMA: Homeostasis Model Assessment of Insulin Resistance (insuline(mUI/l) x glycémie(mmol/l) /22.5)

^f^ eGFR: estimated glomerular filtration rate (using Cockroft and Gault formula (male: 1.23 x weight (kg) x (140-age) / creatinine (μmol/l) and female: 1.04 x weight (kg) x (140-age) / creatinine (μmol/l))

### Changes in bone quality on second line antiretroviral treatment

**Fig 1:**
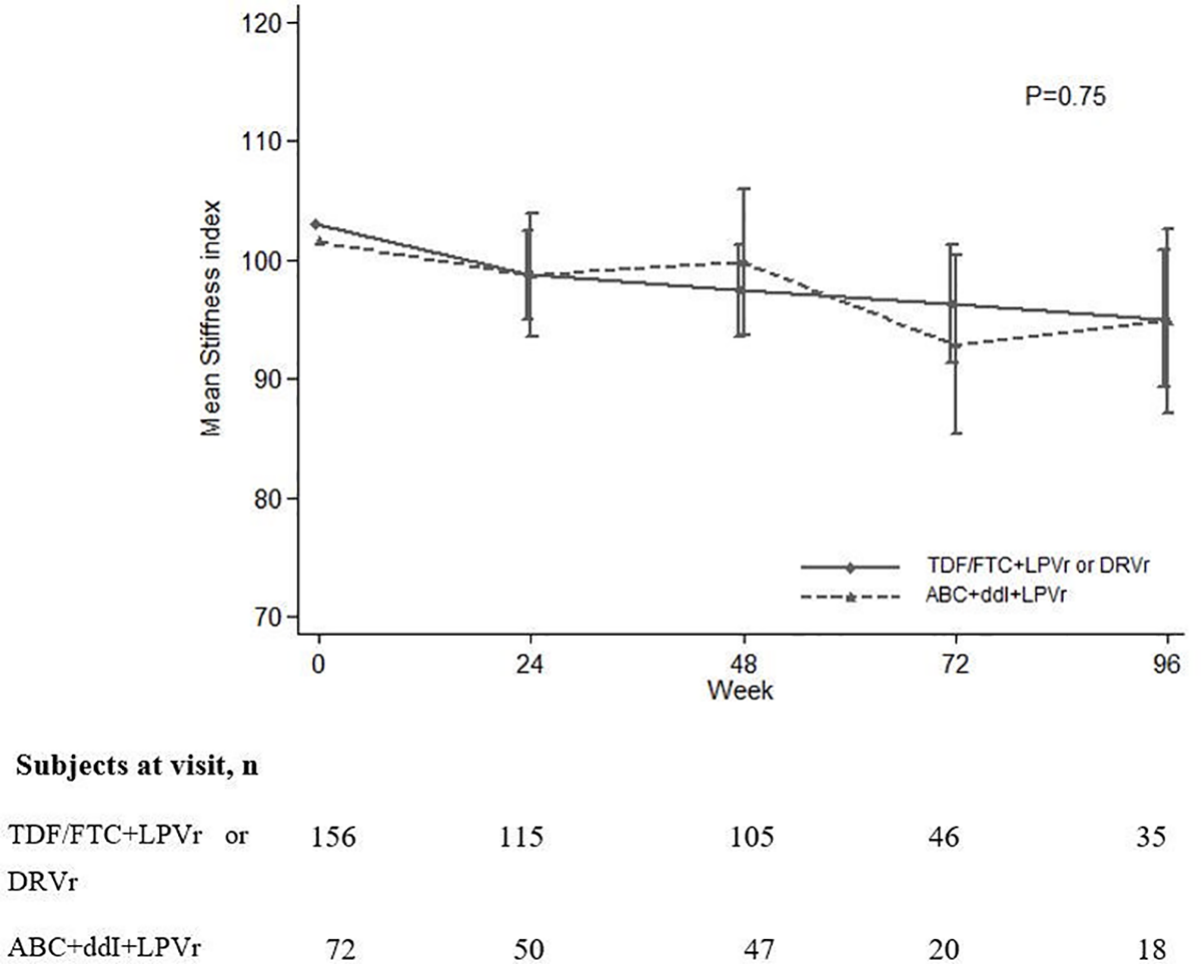
Evolution of bone quality by treatment received. Bone Quality was assessed by calcaneal quantitative ultrasound Stiffness index (Achilles, GE Healthcare, Little Chalfont, UK).

Over 96 weeks of follow-up, 664 SI assessments were realized. After 24, 48 and 96 weeks of second-line ART, the overall mean SI decreased at these points in time by minus 3.7% [-7.6%, 0.4%], minus 4.2% [-8.3%, 0.1%] and minus 7.1% [-13.1%, -1.3%] from its baseline value, respectively, but ultimately there were no difference observed in bone loss between the TDF-based and the TDF-sparing treatment arms (P = 0.75) **(Fig 1)**.

### Risk factors associated with decline in bone quality on second line antiretroviral treatment

Interaction terms combining follow-up duration with the different covariates were not statistically significant, indicating that none of the covariates, including TDF exposure, were found to modify bone loss throughout time. Factors associated with overall levels of bone quality were time of follow-up, age, sex, baseline BMI, and study site. Duration of first-line ART was associated with bone quality for women, and tended to be associated with bone quality for all participants **(Table 4)**. Women in menopause as well as women receiving a Depo-Provera®-based (Pfizer, New York, NY, USA) contraceptive tended to have a lower bone quality than did other women with borderline statistical significance.

**Table 4:** Factors associated with change in Stiffness index during second-line antiretroviral treatment in multivariable analysis.

	All participants			Women		
	B	95% CI	p-value	β	95% CI	p-value
***Demographic factors***						
Follow-up (per 24 weeks)	-0.9	[-1.4; -0.4]	0.001	-1.3	[-2.0; -0.6]	<0.001
Age (per 10 years)	-6.8	[-9.4; -4.1]	<0.001	-5.8	[-9.9; -1.8]	0.005
Female	-9.6	[-15.5; -8.8]	0.001			
Cameroun ^a^	10.9	[5.7; 16.1]	<0.001	10.7	[4.8; 16.5]	<0.001
Year of inclusion in METABODY (2011 vs 2012)	2.3	[-2.6; 7.2]	0.35	1.2	[-4.3; 6.6]	0.68
***Women related factors***						
Menopause				-7.8	[-16.4; 0.8]	0.08
Current Depo-provera using				-3.6	[-7.4; 0.10]	0.06
***HIV infection related factors***						
First-line ART duration (months)			0.07			0.01
< 36	Ref			Ref		
[36–72[	6.4	[0.6; 12.2]		10.2	[3.5; 16.8]	
≥ 72	2.5	[-4.4; 9.3]		5.5	[-2.3; 13.4]	
TDF exposure	1.7	[-4.1; 7.4]	0.57	1.4	[-5.1; 7.8]	0.68
LPV exposure	1.6	[-4.0; 7.2]	0.57	1.1	[-5.1; 7.2]	0.74
***Others factors***						
BMI (kg/m^2^)	0.8	[0.3; 1.4]	0.004	0.6	[0.0; 1.3]	0.039
Regular physical activity ^b^	-1.0	[-2.5; 0.6]	0.23			

TDF: Tenofovir disoproxil fumarate; LPV: Lopinavir; ART: antiretroviral treatment; BMI: body mass index (mass(kg)/height^2^(m)); ART: antiretroviral treatment

^a^ Cameroun (Cameroun vs Burkina Faso and Senegal)

^b^ Regular physical activity: moderate physical activity every day or high physical activity twice a week.

## Discussion

The aim of our study was to describe, among 228 African patients failing first-line ART, the change in bone quality during 96 weeks of follow-up after second-line treatment initiation and to identify associated factors, including the effects of TDF. Second-line ART initiation was associated with 4–7% loss in SI over 48 to 96 weeks of follow-up. HIV infection-related factors or TDF-including regimens were not associated with SI levels or changes over time in our study. Factors associated with greater BMD were younger age, male sex, higher BMI and the Cameroon study site.

In this study, bone quality was assessed by QUS, as an alternative to the standard reference method, DXA, which is not available in many African settings. QUS and DXA devices measure different bone properties, bone quality (including bone density, bone structure and bone elastic properties) and bone quantity (BMD), respectively [24]. Studies comparing these two technologies have shown overall moderate correlations between QUS and DXA parameters, with some variations by body sites and devices used [25,26]. In a study evaluating specifically the Achilles device for osteoporosis diagnosis, the sensitivity for QUS versus DXA ranged from 76% to 84% at repeated measurements during 0–7 years and the specificity ranged from 36% to 57% [27]. Although, QUS and DXA cannot be directly compared, several studies conducted in large groups of healthy subjects have shown that QUS is as effective as DXA in the prediction of fracture risk [28,29]. Yet, to date QUS technology is not recommended by the World Health Organization nor by the International Society for Clinical Densitometry (ISCD) as a means to diagnose osteoporosis because there is no consensus on the type of QUS device that should be used, measured variables, or cutoffs for osteoporosis diagnosis. As a consequence, very limited data is available regarding the prevalence of osteoporosis in sub-African settings [30,31]. Calcaneal QUS can, however, be used as a screening tool to identify people at high risk, based on device-specific cutoffs that are pre-validated in a specific population [32]. The ISCD endorses the use of calcaneal QUS for the prediction of osteoporotic fractures in postmenopausal women and recommends the initiation of preventive treatment if the risk of fracture estimated by calcaneal QUS and clinical features is very high [19]. This technology can also be used safely to monitor changes in bone quality at the individual level and to identify factors associated with bone deterioration in research settings.

Our findings are consistent with a previous study based on DXA scan showing accelerated bone loss after second line treatment initiation [33]. It confirms that the impact of ART on bone is not limited to first treatment introduction, but can also be observed with second-line initiation in patients failing standard first line ART. As ART-related bone loss is poorly reversible, these bone degradations may cumulate over time and successive treatments increase the risk of fractures. In our study, the percentage of bone deterioration at 96 weeks (-7.1%) was marginally higher than the usually reported percentage standing between 2 and 4% at 96 weeks, however the use of different technologies precludes relevant comparison of the magnitude of the effect [8,11,33 34].

In contrast with several first-line randomized trials that compared TDF/FTC to ABC/3TC, Raltegravir or to AZT/3TC, the effect of TDF on bone loss was not confirmed in the present study, although it was powerful enough to detect a difference of about 0.2 standard deviation between the treatment groups [11–13,35]. These discrepancies could be related to the use of a QUS device, instead of the use of osteodensitometry by DXA as these technologies measure different bone properties. Yet, out 3 studies [10,36,37] assessing the association between ART and QUS bone quality, only one showed an association between QUS stiffness index and current use of TDF [36]. Another hypothesis for the lack of TDF effect could be the presence of ddI in the TDF-sparing regimen, as an association between low BMD and ddI exposure was reported in the Nutrition for Healthy Living Study (NFHL) [38].

Other HIV-related factors such as CD4 count, viral load, and WHO clinical stage were not associated with SI at baseline or during administration of second-line ART. Many *in vitro* studies have shown the detrimental role of viral proteins on bone metabolism, but the majority of studies on HIV-positive populations did not find significant differences in bone quality according to viral load [8,9,39–42]. Furthermore, only a few studies have reported a lower BMD in patients with a high viral load [33,43]. The sole HIV-related factor associated with bone quality, in our study, was first-line ART duration. Women with first-line duration between 36 and 72 months had higher SI than women with duration below 36 or above 72 months. We have no explanation for this result given that these women had similar demographic and clinical characteristics.

In contrast, as expected, osteoporosis traditional risk factors like age, gender and BMI were found to be associated with bone quality in our study. We also found marked differences in bone quality between study sites. Average SI in Yaoundé (Cameroon) was higher than those of Bobo Dioulasso (Burkina Faso) and Dakar (Senegal). Genetic and environmental factors could explain this differences in bone quality.

Women receiving a medroxyprogesterone-based (Depo-Provera®, Pfizer, New York, NY, USA) contraceptive tend to have lower bone quality than other women. The effects of medroxyprogesterone on BMD are well-known and have been widely described [44,45]. The use of Depo-Provera® (Pfizer, New York, NY, USA) was proposed early in the study to non-menopausal women, but its use was quickly dropped because of associated side effects (e.g., amenorrhea and dysfunctional uterine bleeding). Only 23 doses were administered to 20 women in the first year. This short-term use may explain why the observed effect was weak.

Regarding the relationship between vitamin D levels and bone quality, it is very controversial, but most studies do not find a difference in BMD between people who are deficient in 25-OH vitamin D as compared with those that are not; this lack of difference is found both in people infected with HIV and in the general population [46–48]. As such, despite its central role in bone metabolism, 25-OH vitamin D was not associated with bone quality in our study at baseline or during follow-up [49]. In the SUN study in the United States, the overall prevalence of 25-OH vitamin D deficiency was 70.3% [50]. In the United Kingdom, there was an average 25-OH vitamin D level of 15.3 (SD = 11) ng/ml and a prevalence of 25-OH vitamin D deficiency of 78.7% [46]. Limited data on vitamin D levels in adults infected with HIV in Africa exists, however. A study in Uganda reported 35% of 25-OH vitamin D deficiency in patients with HIV infection [51]. The average 25-OH vitamin D was 34.1 (SD = 13.9) ng/ml in the general population in Guinea Bissau, with 37% of men and 41% of women having a 25-OH vitamin D deficiency [52]. The prevalence of 25-OH vitamin D deficiency in our study (34.6%) is similar to those found in other studies in sub-Saharan Africa, but lower than those observed in European and North American cohorts [51–53].

Our study has several limitations. First, we recorded a great number of participants lost to follow-up, especially after week 48. Second, bone changes were assessed over only 96 weeks of follow-up. Hence, further investigation with a longer duration of follow-up is needed to assess whether the rapid bone deterioration observed in the two first years is sustained over time. Longer-term follow-up on older participants is also needed to better understand the clinical impacts of our findings. Third, we were not able to assess osteoporosis prevalence due to the inappropriateness of QUS use for this diagnosis.

## Conclusion

Following 96 weeks on second-line ART in African patients, including a majority of women, mean SI decreased by minus 7% as compared with baseline. Factors associated with bone quality included age, BMI, sex, and study site. The effect of TDF on bone loss was not confirmed in this study. These results show that calcaneal QUS can be used to monitor changes in bone quality over time in this population. Further investigations are needed, however, to better understand the possible clinical implications of the QUS bone quality changes that were described in our study. The low level of vitamin D deficiency in our cohort, unlike in European and American cohorts, also deserves to be confirmed.
